# Cultural differences in Psychosis: Breaking the borders of normality

**DOI:** 10.1192/j.eurpsy.2026.11461

**Published:** 2026-07-31

**Authors:** M. Baptista, C. Sequeira, M. Bajouco, S. Morais

**Affiliations:** 1 Psychiatry, Local Health Unit of Coimbra; 2 Faculty of Medicine, University of Coimbra, Coimbra, Portugal

## Abstract

**Introduction:**

According to the 2023 Migration and Asylum Report from *Agência para Integração Migrações e Asilo* (AIMA), the foreign population in Portugal increased by 33.6% compared to the previous year. The most frequent nationalities include Brazil, Angola, Cape Verde, the United Kingdom, and India. Migration is well established as a risk factor for psychosis. Importantly, literature suggests that 15–30% of psychotic symptoms are shaped by the patient’s cultural background.

**Objectives:**

To explore cultural variations in psychosis phenotypes.

**Methods:**

Clinical case report and literature review. Searches were conducted in *PubMed* and other databases using keywords such as *migration*, *phenotypes*, and *psychosis.*

**Results:**

A 35-year-old Nepali male, living in Portugal for two years and employed as a builder, was admitted after a suicide attempt. During hospitalization, he presented persecutory delusions towards his flatmates and auditory hallucinations in the form of laughter. He was diagnosed with schizophrenia and discharged on intramuscular risperidone 100 mg monthly and oral lorazepam 2.5 mg. One month later, he was readmitted with recurrence of psychotic symptoms, now including mystic delusions and behavioural disorganization (e.g., staying undressed in the hospital chapel). According to his flatmate, he had not been religious before. Risperidone was rescheduled to 21-day intervals, and haloperidol 10 mg was added, with no improvement. Communication was hindered by the linguistic barrier—he spoke only Nepali—and there was a constant struggle to distinguish pathology from culturally comprehensible behaviour.

**Conclusions:**

There is an important ethnical variability in the expression and content of psychotic symptoms influenced by social and cultural norms of a specific society, but also, as recent research shows, by genetic differences between populations. Moreover, many of the diagnostic tools we have available were developed in Western societies, meaning that defined symptoms of psychosis are deeply linked to the cultural background of Western psychiatry, and so the way we evaluate patients from other cultural backgrounds might not be the most adequate. Psychosis should therefore not be treated as a homogeneous entity; cultural background must be carefully considered in both diagnosis and management.

**Disclosure of Interest:**

None Declared

